# A Rare Case of Delayed Onset Multi-Drug Interaction Resulting in Rhabdomyolysis in a 66-Year-Old Male

**DOI:** 10.7759/cureus.20035

**Published:** 2021-11-30

**Authors:** Harmony Osborn, Daniel Grossman, Smriti Kochhar, Anish Kanukuntla, Priyaranjan Kata, Pramil Cheriyath

**Affiliations:** 1 Internal Medicine, Hackensack Meridian Health Ocean Medical Center, Brick, USA

**Keywords:** acute kidney injury, ticagrelor, rosuvastatin, amiodarone, drug-drug interactions, rhabdomyolysis

## Abstract

Drug-drug interactions in medications metabolized by cytochrome P450 enzymes can lead to multi-organ complications. An uncommon but serious potential adverse effect of statins is rhabdomyolysis, most commonly triggered by drug interactions. Rhabdomyolysis presents with markedly elevated creatine kinase levels, dark urine, and often myoglobinuria. The breakdown of the muscles during rhabdomyolysis can be toxic to the kidneys, often precipitating acute kidney injury (AKI) and can also damage the liver, causing transaminitis. This study presents a case of a 66-year-old male with delayed onset complex pharmacological interaction between ticagrelor, rosuvastatin, and amiodarone resulting in rhabdomyolysis, AKI, and transaminitis.

## Introduction

Polypharmacy has been associated with adverse outcomes such as an increased risk of adverse drug events, drug-drug interaction, and multiple geriatric syndromes. These risks are especially prevalent in individuals taking more than five medications [1]. Patients taking statins as part of polypharmacological regimen benefit from regular monitoring of creatine kinase and liver enzymes compared to initial measurement prior to statin initiation. Drug-drug interactions have been commonly documented between rosuvastatin and amiodarone resulting in transaminitis and rhabdomyolysis [2,3]. Rare reactions between ticagrelor and rosuvastatin have also resulted in rhabdomyolysis in as many as seven documented cases [4]. Rhabdomyolysis is characterized by markedly elevated creatine kinase levels, muscle pain, and myoglobinuria. The severity of rhabdomyolysis can range from isolated elevation in muscle enzymes to life-threatening electrolyte imbalance and acute kidney injury (AKI) [3]. The focus of this case study is to analyze the interaction of multiple medications including amiodarone, rosuvastatin, and ticagrelor which likely triggered the onset of rhabdomyolysis in a 66-year-old male who had been taking the above medications for an eight-month period of time.

## Case presentation

A 66-year-old Caucasian male presented to the ER reporting blood in the urine with increased fatigue for the past three days. He explained that the urine was slightly red and progressively worsened to gross bloody hematuria. The patient had been on aspirin and ticagrelor (DAPT therapy) for eight months prior to hospitalization immediately following cardiac stenting for acute coronary syndrome. The patient was also taking rivaroxaban (15 mg) and amiodarone (200 mg) for paroxysmal atrial fibrillation. The rivaroxaban was stopped five months prior to the current hospital admission due to epistaxis. The patient reported that he had started calisthenic exercises for the past two months prior to presentation. He had been sore occasionally following these exercises. On review of symptoms, the patient denied dysuria, abdominal pain, flank pain, fever, trauma, or injury. His past medical history was significant for myocardial infarction, paroxysmal atrial fibrillation, hypertension, and hypercholesterolemia. His medication regimen included amiodarone 200 mg once a day, Rosuvastatin 20 mg every evening, ticagrelor 90 mg every 12 hours, aspirin 81 mg once a day, metoprolol tartrate 50 mg every 12 hours, pantoprazole 40 mg every morning before breakfast, and alprazolam 1 mg as needed for anxiety.

**Figure 1:**
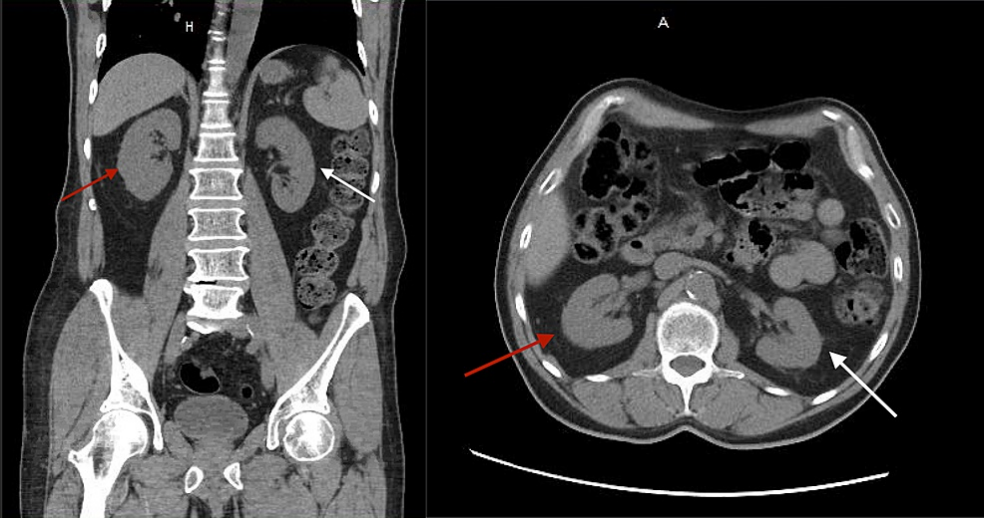
Non-contrast CT coronal view (A), axial view (B) of the abdomen. Figures 1A and 1B show right (red arrow) and left (white arrow) kidneys with no nephrolithiasis or perinephric fat.

The only medication that was new to his regimen was the alprazolam, prescribed for anxiety two months prior to presentation. The patient had been adherent to his medication regimen for the past eight months. He denied any alcohol, tobacco, or recreational drug use. Vitals on admission were blood pressure 164/74 mmHg, pulse 71 beats per minute, respiratory rate of 20 respirations per minute, temperature 97.9 degrees Fahrenheit and pulse oximetry of 98% on room air. His initial labs were significant for low RBC count and platelets at 127,000/mm3. Differential analysis showed slight anisocytosis, macrocytes, ovalocytes, and abnormal RBC morphology. Complete metabolic panel showed an increased creatinine of 1.70 mg/dL (with a glomerular filtration rate [GFR] of 41 ml/min), alkaline phosphatase of 232 u/L, aspartate aminotransferase (AST) of 1,290 U/L, and alanine aminotransferase (ALT) of 766 U/L. The creatine kinase measured two days after admission was 33,030 U/L. The patient’s hepatitis panel, coagulation panel, thyroid panel, and acetaminophen level were all within normal limits. Urinalysis showed proteinuria with 100 mg/dl, a few squamous epithelial cells, large amounts of blood with few RBCs, and 3-5 WBCs, without leukocytosis. A CT scan of the abdomen and pelvis without contrast showed no nephrolithiasis or appendicitis (Figure 1). The ultrasound of the right upper quadrant showed no pathological findings of the kidney or the liver.

On admission, amiodarone and rosuvastatin were stopped due to the patient’s transaminitis. IV hydration therapy was started on admission due to the AKI. Later on in the patient’s course of treatment, after rhabdomyolysis was diagnosed, anti-Jo antibodies and antinuclear antibodies were measured and found to be negative. On the day the patient was discharged, five days after the initial encounter, the patient’s condition had progressively improved, which was evidenced by the patient’s level of serum creatine improving to 1.28 mg/dL (with a GFR of 56 ml/min), alkaline phosphatase of 215 U/L, AST of 614 U/L, ALT of 650 U/L and a creatine kinase of 10,297. The patient's admission and discharge day's laboratory values are listed in Table 1.

**Table 1:** Laboratory values. BUN: Blood urea nitrogen; AG ratio: Albumin Globulin ratio; eGFR: Estimated glomerular filtration rate; AST: Aspartate aminotransferase; ALT: Alanine transaminase.

Lab Test	Admission day	Discharge day	Normal values
WBCs	5.9 x 10*^3^/uL	4. 9 x 10*^3^/uL	4.5-11.0 x10*^3^/uL
RBCs	3.76 x10*^6^/uL	3.53 x 10*^6^/uL	4.50-5.30 x10*^6^/uL
Hemoglobin	11.9 g/dL	11.3 g/dL	12.0-17.5 g/dL
Hematocrit	35.3%	32.9%	36.0-53.0%
Glucose	90 mg/dL	167 mg/dL	70-99 mg/dL
BUN	17 mg/dL	14 mg/dL	5-25 mg/dL
Creatinine	1.70 mg/dL	1.28 mg/dL	0.61-1.24 mg/dL
Albumin	3.7 g/dL	2.8 g/dL	3.5-5.0 g/dL
eGFR	41 mL/min/1.73m*^2^	56 mL/min/1.73m*^2^	>60 mL/min/1.73m*2
Sodium	137 mmol/L	138 mmol/L	136-145 mmol/L
Potassium	4.2 mmol/L	3.5 mmol/L	3.5-5.2 mmol/L
Phosphorus	2.2	2.1	2.5-4.6 mg/dL
Magnesium	2.0	2.0	1.3-2.5 mg/dL
Chloride	106 mmol/L	112 mmol/L	96-110 mmol/L
Anion Gap	6.0 mmol/L	5.0 mmol/L	5-13 mmol/L
Calcium	9.0 mmol/L	8.3 mmol/L	8.5-10.5 mg/dL
Carbon Dioxide	25 mmol/L	21 mmol/L	24-31 mmol/L
Alkaline Phosphatase	232 U/L	215 U/L	38-126 U/L
Protein Total	7.0 g/dL	5.7 g/dL	6.0-8.0 g/dL
AG Ratio	1.1	1.0	>1.0
Bilirubin Total	1.1 mg/dL	0.7 mg/dL	0.0-1.3 mg/dL
AST	1,290 U/L	614 U/L	10-42 U/L
ALT	766 U/L	650 U/L	10-60 U/L
Creatine Kinase	33,030 U/L	10,297 U/L	22-232 U/L

## Discussion

Polypharmacy in patients with multiple co-morbidities can cause fatal drug-drug interactions [4]. The prevalence of rhabdomyolysis in patients on rosuvastatin is 1-3% [5]. Although myopathy/rhabdomyolysis is a well-documented adverse effect of statin therapy, a literature search had revealed only a few studies regarding the drug interaction between ticagrelor, rosuvastatin, and amiodarone, resulting in rhabdomyolysis [5]. The drug-drug interaction of ticagrelor and rosuvastatin, the interaction of amiodarone and rosuvastatin, as well as complicating factors such as preexisting nephropathy in relation to rhabdomyolysis will be discussed here in relation to this patient's case.

The concurrent usage of rosuvastatin and the antiarrhythmic amiodarone is known to increase the risk of myopathy, hepatotoxicity, and rhabdomyolysis [3]. Rosuvastatin is an enzyme 3-hydroxy-3-methyl-glutaryl-coenzyme A (HMG-CoA) reductase inhibitor metabolized by both hepatic enzyme CYP2C9 and the renal system. Statins use other pathways for elimination from the body including the permeability glycoprotein 1 (P-gp), multidrug resistance protein 1 (MDR1), and CD243 [5,6]. Amiodarone is a class III antiarrhythmic agent that undergoes metabolism via CYP2C8 and CYP3A4 and inhibits CYP450 isoenzymes 1A2, 2C9, 2D6, and 3A4. Simultaneous use of amiodarone and statins causes an excess in serum levels of statins due to the inhibitory effect of amiodarone on the metabolizing enzymes required for the metabolism of statins. Similar to amiodarone, ticagrelor also inhibits the CYP3A4 enzyme and acts as a substrate for CYP3A4 to be converted to its active metabolite [6]. In this patient, ticagrelor, rosuvastatin, and amiodarone were medications that were well tolerated for up to eight months. Statin-induced rhabdomyolysis can occur anytime during the course of therapy but is most likely to occur within the first six months on average [7].

This patient was concomitantly exposed to ticagrelor and rosuvastatin for eight months. Although there is no direct interaction that has been documented between the two drugs, a similar presentation in a 70-year-old male after 1.5 years of concomitant use of rosuvastatin and ticagrelor with rhabdomyolysis has been presented by Vrkić Kirhmajer M et al. in a case series [4]. There are limited publications on this interaction and the exact mechanism has not been established. This interaction may be due to renal impairment caused by ticagrelor, leading to decreased renal excretion of rosuvastatin. In patients on ticagrelor, serum creatinine concentration was increased by more than 30% in 25.5% of the population [4]. Renal impairment in patients on ticagrelor has been more commonly documented in patients over the age of 75 on angiotensin-converting enzyme (ACE) or angiotensin receptor blocker (ARB) inhibitors [4]. An earlier occurrence of renal impairment could be due to the concurrent use of amiodarone with other medications. Ticagrelor competes on a transporter level (OATP1B1, P-glycoprotein, ABCG2, MRP2), leading to decreased biliary and renal excretion of rosuvastatin which also has added to drug-drug interaction with other P-gp substrates (in our case rosuvastatin and amiodarone) [4-6].

This patient presented to the ER with AKI which may have triggered rhabdomyolysis by decreased excretion of rosuvastatin. Age-related decline of kidney function, dehydration, or possibly hypertension-related kidney injury may have been possible in this patient, inciting the kidney injury and resultant drug toxicity. However, it is more likely that drug toxicity resulting in rhabdomyolysis caused an AKI. This patient's CK levels were first titrated after two days of hospitalization which delayed the official diagnosis of rhabdomyolysis. It is likely that rhabdomyolysis caused the AKI by a combination of mechanisms such as hypovolemia, intraluminal obstruction by myoglobin, uric acid casts, direct myoglobin toxicity, renal ischemia secondary to muscular vasoconstrictors, and production of free radicals [8]. Delayed diagnosis of rhabdomyolysis can be fatal due to electrolyte imbalance, kidney and liver damage. In this case, the patient’s amiodarone and rosuvastatin were withheld on admission and the patient was given IV fluid despite the delay in making the diagnosis of rhabdomyolysis. While it would have been better to have measured the CK earlier to have gotten the diagnosis earlier, the patient did receive the right treatment for rhabdomyolysis from the start of his admission, even before the disease was diagnosed by taking a CK level.

While the last medication that was added in the case of this patient was alprazolam, two months before his admission, there is no correlation in the literature suggesting an interaction between alprazolam and ticagrelor or rosuvastatin independently. Alprazolam is also a substrate for CYP3A4 which may have decreased the metabolism of both ticagrelor and amiodarone. The combination of these three drugs may have increased the level of rosuvastatin resulting in rhabdomyolysis [5,9].

This outcome may have been prevented by decreasing the number of medications the patient was taking or limiting new medication initiation. Using non CYP450 statins in patients who are on more than five related drugs is recommended to prevent adverse drug effects. In patients taking ticagrelor, CK and renal function should be regularly monitored [10]. Genetic variability in drug metabolism alters patient susceptibility to myopathy, the most common adverse statin effect. Patients may benefit from genetic testing for statin-induced myopathy prior to drug initiation [5]. In treating patients with rhabdomyolysis, the first practice should be to identify and stop the initial precipitating factor which can be accomplished by taking a thorough history, analysis of complete blood work with comprehensive metabolic panel, CK levels, urine analysis, kidney function, complete blood count and immunologic and viral serologies. In this case, the patient’s dark urine and very high CK level (much higher than five times the upper limit of normal CK) were enough to make the diagnosis of rhabdomyolysis.

In cases of polypharmacy and statin use, patients are at a higher risk of myopathy when they participate in the exercise. Statins cause higher rates of muscle cell turnover and in addition to the physical stress of exercise, muscle damage is more likely. Rhabdomyolysis can be exacerbated by continued exercise if not recognized [11]. This patient had started a calisthenic regimen in the two months prior to his admission and it likely contributed to his case of rhabdomyolysis.

## Conclusions

The risk of developing statin-induced rhabdomyolysis is dependent on the blood concentration of the statin. When there is any decline in renal function, either as a result of ticagrelor or other physiologic changes, there is a significant risk of incurring rosuvastatin-related rhabdomyolysis. Polypharmacy of ticagrelor, amiodarone, and rosuvastatin should either be avoided in patients with increased risk of renal injury or should warrant increased clinician vigilance. Rhabdomyolysis may occur any time during rosuvastatin therapy. Surveillance should be considered in regards to patient kidney function and serum CK to prevent poor outcomes in older populations.
